# Medicinal plants potential and use by pastoral and agro-pastoral communities in Erer Valley of Babile Wereda, Eastern Ethiopia

**DOI:** 10.1186/1746-4269-8-42

**Published:** 2012-10-22

**Authors:** Anteneh Belayneh, Zemede Asfaw, Sebsebe Demissew, Negussie F Bussa

**Affiliations:** 1 Biology Department, Haramaya University, P.O. Box 282, Haramaya, Ethiopia; 2 Department of Plant Biology & Biodiversity Management, National Herbarium, Addis Ababa University, P.O. Box 3434, Addis Ababa, Ethiopia; 3 School of Medicine, Haramaya University, P.O. Box 138, Haramaya, Ethiopia

**Keywords:** Babile, Erer Valley, Ethnomedicinal plants, Pastoralists, Agro-pastoralists, Eastern Ethiopia

## Abstract

**Background:**

Ethiopian plants have shown remarkably effective medicinal values for many human and livestock ailments. Some research results are found on medicinal plants of the south, south west, central, north and north western parts of Ethiopia. However, there is lack of data that quantitatively assesses the resource potential and the indigenous knowledge on use and management of medicinal plants in eastern Ethiopia. The main thrust of the present ethnobotanical study centres around the potential and use of traditional medicinal plants by pastoral and agro-pastoral communities in Babile Wereda (district) of eastern Ethiopia. The results can be used for setting up of conservation priorities, preservation of local biocultural knowledge with sustainable use and development of the resource.

**Materials and methods:**

Fifty systematically selected informants including fifteen traditional herbalists (as key informants) participated in the study. Semi-structured interviews, discussions and guided field walk constituted the main data collection methods. Techniques of preference ranking, factor of informant consensus and Spearman rank correlation test were employed in data analysis. Medicinal plant specimens were collected, identified and kept at the National Herbarium (ETH) of Addis Ababa University and Haramaya University Herbarium.

**Results:**

Fifty-one traditional medicinal plant species in 39 genera and 28 families were recorded, constituting 37% shrubs, 29% trees, 26% herbs, 6% climbers and 2% root parasites. Leaves contributed to 35.3% of the preparations, roots (18.8%) and lower proportions for other parts. Formulations recorded added to 133 remedies for 54 human ailments, in addition to some used in vector control. The majority of remedies were the juice of single species, mixtures being generally infrequent. *Aloe pirottae*, *Azadirachta indica* and *Hydnora johannis* were the most cited and preferred species. *Aloe pirottae,* a species endemic to Ethiopia, is valued as a remedy for malaria, tropical ulcer, gastro-intestinal parasites, gallstone, eye diseases and snake bite. The jel extracted from dried and ground plant material, called SIBRI (Oromo language), was acclaimed as a cleaner of the human colon. Concoction made from leaf, seed and flower of *Azadirachta indica* was given for treatment of malaria, fungal infections and intestinal worms. Root preparations from *Hydnora johannis* were prescribed as remedy for diarrhoea, haemorrhage, wound and painful body swelling, locally called GOFLA (Oromo language).

**Conclusions:**

The study documented many well known and effective medicinal species of relevance for human healthcare, including for the treatment of malaria which is rampant in the area as it is in many parts of Ethiopia. This underscores the importance of the traditional medicinal plants for the people living in the area and the potential of the resource for development. Consequently, the study area deserves urgent conservation priority coupled with mechanisms for the protection of the associated indigenous medical lore as well as development and effective use of the medicinal plant resource.

## Introduction

Ethiopian plants have shown remarkably effective medicinal values for many ailments that affect people and livestock. Knowledge of the medicinal plants of Ethiopia and of their uses provides a vital contribution to human and livestock healthcare needs throughout the country [1-5]. About 80% of the Ethiopian population is said to depend on traditional medicine for their healthcare delivery and most of this comes from plants [6]. Medicinal plants found in the Ethiopian flora were at one time estimated to be over 700 species [7] while in a later communication [8] about 1000 identified species were included. About 300 of the traditional medicinal plant species of Ethiopia are frequently mentioned in many sources [4,8-13]. Many other medicinal plant of Ethiopia mainly found in lesser studied areas still awaits scientific studies.

A review of the main sources shows that studies on medicinal plants of Ethiopia have so far concentrated in the south, southwest, central, north and north western parts of the country [1-4,6,8,10,13-17]. Data that quantitatively assess the resource potential, indigenous knowledge on the use and management of medicinal plant species from eastern Ethiopia and specifically from the present study area are lacking. The pastoral and agro-pastoral communities of Ethiopia are largely found in the eastern, south eastern and north eastern parts of the country, i. e. the Somali, Oromo and Afar pastoralists constitute 87 percent of the total pastoralist population in Ethiopia [18]. The traditional medicinal plant lore and potentials have not been investigated to a noticeable level. Similar to many other rural communities in Ethiopia, the use of traditional medicinal plants plays a vital role in human and livestock healthcare systems in the pastoral and agro-pastoral communities of Babile and its surroundings. This is so because modern health services, which are mostly unaffordable to most people, are also very limited in their coverage while on the other hand there is general acceptance of the traditional herbal medical system by the community. As the local communities encounter cultural changes due to the current development activities in areas where these communities reside, unless the plants are conserved and the ethnomedicinal knowledge is documented, both the medicinal plants and the associated indigenous knowledge of the people could vanish forever.

The Oromo and the Somali people living in the upper and lower Erer Valley of the Babile Wereda (district) are expected to be the custodians of valuable indigenous knowledge on the use of traditional medicinal plants of their surroundings, which they use for treating human and livestock ailments. Access to modern health services for both humans and livestock is very limited. The dependence of the local medical system on the use of traditional medicinal plants could partly be attributed to the underdeveloped nature of the modern medical system in the general area. Given the diversity of higher plant species in the area, about 238 species documented from the Erer Valley [19], the share of medicinal plants and the value of the associated indigenous knowledge of the pastoral and agro-pastoral communities is expected to be high.

However, the pastoral and agro-pastoral communities in this area have remained ethnobotanically unexplored and there is no comprehensive account of the traditional medicinal practices. Therefore, the objective of this study was to (1) assess and document the medicinal plant potential; (2) investigate and gather information on indigenous knowledge and use of medicinal plants by the local communities; (3) indicate the possible ways for implementation of proper management, conservation and sustainable use of the medicinal plants and the natural vegetation of the study area.

## Materials and methods

### Study area

**Figure 1:**
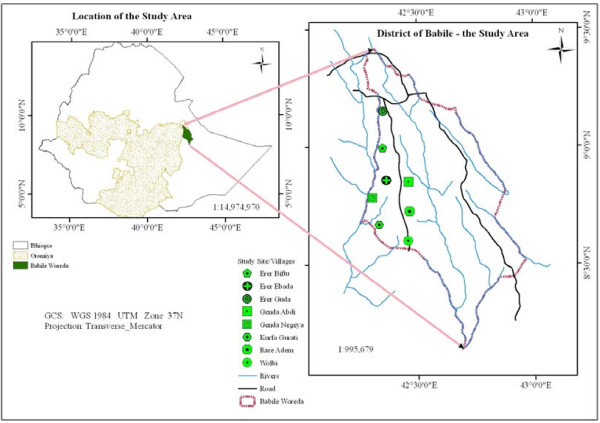
Location map of the study area.

The study area, Erer Valley (upper Erer and lower Erer) of Babile Wereda in the eastern lowlands of Ethiopia, is situated at the semi-arid trans-boundary of Oromia and Harari Regions, located at about 560 km southeast of Addis Ababa. It is delimited with coordinates of latitudes 08^o^22'20" - 09^o^30'30"N and longitudes 42^o^20'10" - 42^o^30'50"E and its elevation ranges between 940 m and 1585 m a.s.l. (Figure 1). The lower Erer valley is part of the protected area for the African elephant (*Loxodonta africana*) known as Babile Elephant Sanctuary (BES).

There is a high degree of competition for space and food between elephants and large herds of cattle and camels in the area [19]. There is also an indication of the population increment in the last 15 or more years, resulting in a high density of human settlement in the Erer Valley. The human population in the rural part of Babile Wereda (district), which mostly covers the Erer Valley, is about 75,970 (38,371 males and 37,599 females) [20].

The vegetation of the valley belongs to the *Acacia-Commiphora* woodland, semi-desert scrubland and evergreen scrub types. There are also riverine woody species in the upper Erer Valley that include species of *Acacia robusta* Burch.*, Tamarindus indica* L.*, Oncoba spinosa* Forssk.*, Acokanthera schimperi* (A. DC.) Schweinf. and *Capparis tomentosa* Lam. [21].

The mean annual temperature is about 18.2°C, ranging from a mean minimum of 10.8°C to mean maximum of 29.4°C. There is only a slight difference in temperature throughout the year, with the hottest months during April to June (maximum 29.6°C) and the coldest during October to December (minimum 8.2°C). The mean annual rainfall is 712.6 mm year^-1^, with high variation from year to year, ranging from 462.6 mm to 1210.4 mm year^-1^. Rainfall is bimodal, occurring from February to April (short rain season) and June to September (long rain season) (Source: National Metrological Service Agency of Ethiopia Data from 1965 to 2005).

### Data collection methods

Ethnobotanical data were collected in two different seasons, from September to November 2009 and June to August 2010. Participatory Rural Appraisal (PRA) techniques were employed to collect data, as recommended by Martin [22] and Cunningham [23]. Eight sampling sites were identified from the study area where settled farmers and trans-human pastoralists were found and indicated on the location map of the study area (Figure 1). Ethnobotanical information was collected from 50 informants (37 male and 13 female). Among the 50 informants, 15 were key informants (traditional healers) selected with the assistance of clan leaders, peasant association leaders and members of the local community. Purposive sampling technique was used for selection of key informants while for the others stratified random sampling was employed. The informants were grouped into three age groups, young (20–35), adult (36–50) and elderly (above 50) to see how the knowledge varies with age.

Before carrying out the interviews and group discussions an oral Prior Informed Consent (PIC) was sought from every respondent. Furthermore, participants collectively endorsed the research by giving oral blessings in their usual traditional style. Semi-structured interviews and group discussions were administered in the local (Oromo and Somali) languages to collect basic information on the Indigenous Knowledge (IK) on the traditional uses of medicinal plant species together with their local names, diseases treated or controlled, part used, conditions and method of preparation, part administered, dosage used and major drawbacks. Further, practical observation sessions and guided field walks with key informants were employed to collect voucher specimens of each medicinal plant species with additional notes. Photographic and video cameras were used for graphic documentation. Most of the interviews were made in the field in order to avoid the risk of confusing identity of plant species by repeated inquiries at least three times with the same and different informants so as to confirm the validity and reliability of the recorded information. Specimens were collected and numbered on the spot, later identified using taxonomic keys in the relevant volumes of the Flora of Ethiopia and Eritrea and through visual comparisons with authenticated plant specimens kept at the National Herbarium (ETH) of Addis Ababa University and at the Herbarium of Haramaya University. The identification was finally confirmed by a senior plant taxonomist and voucher specimens of the medicinal plants deposited at both herbaria.

### Data analysis

Ethnobotanical data were analyzed using both qualitative and quantitative methods following Martin [22] and Cotton [24]. For each medicinal plant, the proportion of informants who independently reported its use against a particular disease/disease category, the informant consensus factor (F_ic_) was calculated using the formula: *F*_*ic*_ = *n*_*ur*_*n*_*t*_/*n*_*ur*_–1 [25,26],

Where, *n*_*ur*_ is the “number of use-reports” in each disease category and *n*_*t*_ is the number of taxa used.

Based on the general informant consensus the preference ranking technique was employed to rank the priority medicinal plants as given by key informants’ preferences indicating the degree of efficacy [22]. In the preference ranking exercise, an integer value (1,2,3,4 and 5) was given, where the most important medicinal plant was given the highest value (5), while the least important is assigned a value of “1”. These numbers were summed for all respondents, giving overall ranks to the medicinal plants. Spearman rank correlation test was run in SPSS 18.00 to analyze the correlation of the informant consensus value and the informant preference ranking value; and binomial test was run in SPSS 18.0 to evaluate the depth of knowledge with age categories in which pair wise age category test was considered. P-value of less than 0.05 was considered a statistically significant difference. MS Excel Spreadsheet was used to generate bar graphs.

## Results and discussion

A total of 51 plant species distributed among 39 genera and 28 families were documented as traditional medicines against human ailments (Table 1). Fabaceae had the highest number of species (13), followed by Capparidaceae, Euphorbiaceae and Tiliaceae each with 3 species; Acanthaceae, Balanitaceae, Lamiaceae, Malvaceae and Meliaceae each with 2 species and the rest 19 families had 1 species each. Out of the total species, 22 were reported by Tamene [27], and 25 species were reported by Tadesse and Demissew [9]. In a similar study by Abbink [10] on medicinal plants of the Me’en people in southwest Ethiopia, an area with a relatively better vegetation cover, 52 species of medicinal plants were reported. In Welenchiti area of Boosat District a total of 81 medicinal plants used by the local people were reported [28]. The number of medicinal plant species reported in this study is considerable, though application of long-term participant observation techniques could add more medicinal plant species to the present list, given the floristic richness and the strongly plant-based biocultural background of the people. The traditional systems and religious beliefs that generally restrict the way of transferring indigenous knowledge might have constrained, to some extent, the free flow of information on medicinal plants in this study.

**Table 1:** List of traditional medicinal plants used for human ailments in Erer Valley

**Voucher No.**	**Scientific name**	**Family**	**Vernacular names**	**Habit**	**Disease treated**	**PU**	**MP**	**PA/MA**
AHU08	*Abutilon fruticosum *Guill. & Perr.	Malvaceae	Balanbal (S)	Sh	Wound	L	Crushed & tied	Dermal
AHU04	*Acacia albida *Del.	Fabaceae	Gerbi (Or, S)*	T	Stomachache/diarrhea Haemorrhage Cough, pneumonia Postpartum complications Kidney disease	B,F,L B,F,L B,F,L B,F,L B,F,L	Concoction Crushed Concoction Concoction Concoction	Oral Anal Oral Oral Oral
AHU122	*Acacia brevispica *Harms	Fabaceae	Hamaresa (Or)*	Sh	Headache	L	Hot infusion	Oral
AHU101	*Acacia nilotica *(L.) Willd. ex Del.	Fabaceae	Serkema (Or)Mere-aga, Galol (S)*	T	Mouth infection Toothache Bad breath (Halitosis) Dysentery Hemorrhoids	L L L & B B B	Crushed Crushed Decoction Concoction Crushed	Chew & spit Chew & spit Herbal bath Oral Anal
AHU03	*Acacia oerfota* (Forssk.) Schweinf.	Fabaceae	Ajo (Or)	Sh	Anal parasitic expel Bad sprit & 'MICH'	StB	Rubbed Crushed & tied	Anal Dermal
AHU1	*Acacia robusta *Burch.	Fabaceae	Wangeyo (Or)	T	Malaria	R	Concoction	Oral
AHU47	*Acacia senegal *(L.) Willd.	Fabaceae	Sobensa (Or),Edad, Adad (S)*	Sh	Eye disease Backbone pain Constipation Stomachache	G G G G	Decoction Fresh gum Fresh gum Fresh gum	Eye bath Eaten raw Eaten raw Eaten raw
AHU48	*Acacia tortilis *(Forssk.) Hayne	Fabaceae	Tadacha, Dhadhacha Or)*	T	Throat infection Stomachache/diarrhea	LB	Concoction Hot infusion	Oral Oral
AHU33	*Acokanthera schimperi *(A. DC.) Schweinf.	Appocynaceae	Kararo (Or), Wabayo, Oboyo (S)*	Sh	Mosquito repellent Malaria Tonsillitis	StL	Burning to smoke Concoction	Smoking the area to stifle the insect Rinsing the throat
AHU13	*Aloe pirottae *Berger ^*♣*^	Aloaceae	Hargeysa (Or), Gebedherta, De’ar (S)	Sh	Tropical ulcer Colon cleaner - SIBRI Eye disease Malaria Snake bite Gallstone Insect repellent	LJ Sa J L L L	Fluid extract Dried & ground Ointment Jel extract Concoction Fluid extract Dried leaves	Dermal Oral Eye Oral Oral Oral Smoking the area to stifle the insect
AHU14	*Asparagus leptocladodius *Chiov.	Asparagaceae	Keleme sere (Or)	Sh	Kidney & liver disease Vomiting of children (Emesis)	LR	Crushed Concoction and Hot infusion	Herbal bath Oral Oral
AHU 10	*Azadirachta indica *A. Juss.	Meliaceae	Kinina (Or)	T	Malaria Fungal infection Intestinal worms	L L OS Fl OS	Leaf extract Leaf extract, and Oil from seed Flower extract, Oil from seed	Oral External Oral
AHU11	*Balanites aegyptiaca *(L.) Del.	Balanitaceae	Bedeno (Or), Got, Kutan (S)**	T	Snake bite Premature ejaculation Influenza Malaria Wound	R F OS R L	Crushed & tied Eaten raw Boiled with tea|*hoja *Concoction Crushed and tie	Dermal Oral Oral Oral External
AHU56	*Balanites glabra *Mildbr. & Schlecht.	Balanitaceae	Kutka (Or)**	T	Fever	R	Crushed	Herbal bath
AHU 15	*Barleria eranthemoides *R. Br. ex C.B. Clarke	Acanthaceae	Kumutu gala (Or), Goda-adosha (S)	H	Infertility of women Rh factor disease	RR	Smoke bath Smoke bath	Smoke bath Smoke bath
AHU 07	*Boscia minimifolia *Chiov.	Capparidaceae	Meygag (S)	T	Wound	L	Crushed & tied	Dermal
AHU 09	*Capparis fascicularis *DC.	Capparidaceae	Hida sere (Or)	Cl	Toothache Wound	R R	Chewed Crushed & tied	Chew & spit Dermal
AHU 25	*Capparis sepiaria *L.	Capparidaceae	Riga gange (Or)	Sh	Swollen body (with oozing pus)	L	Crushed & tied	Dermal
AHU 27	*Cissus rotundifolia *(Forssk.) Vahl	Vitaceae	Shumbur lubu (Or)	Cl	Gonorrhea	L	Concoction	Oral
AHU 32	*Commelina stephaniniana *Chiov.	Commelinaceae	Hola gabis (Or)	H	Skin fungus	St	Cream/sap	Dermal
AHU 59	*Commicarpus sinuatus *Meikle	Nyctaginaceae	Kontom (Or)	H	Throat infection Urination problem	L L	Concoction Concoction	Throat bath Oral
AHU 20	*Commiphora schimperi *(Berg) Engl.	Burseraceae	Dekero (Or) Kobok (S); Hamessa (Or)	T	Treatment of vagina due to birth- Colporrhexis	B	Dried	Smoke bath
AHU41	*Corchorus trilocularis *L.	Tiliaceae	Mulukiya (Or)	H	Abdominal disorder	St	Concoction	Oral
AHU16	*Crotalaria laburnifolia *L.	Fabaceae	Darga, Gelelo (S)	Sh	Skin fungus	L	Crushed	Dermal
AHU17	*Crotalaria retusa *L.	Fabaceae	Bobo-halle (S)	H	Swollen body part- GOFLA	R & L	Concoction	External
AHU18	*Cucumis dipsaceus *Ehrenb. ex Spach	Cucurbitaceae	Hare goge (Or)	H	Snake bite Carnivore bite wound Gallstone, hepatitis	R F R	Crushed Crushed Concoction	Dermal Dermal Oral
AHU26	*Dodonaea angustifolia *L. f.	Sapindaceae	Edechaa (Or)	Sh	Parasitic worms	R	Rubbed	Anal
AHU21	*Euclea racemosa *Murr. ssp. *schimperi *A. DC	Ebenaceae	Mi'esaa (Or)	Sh	Joint pain	L	Hot infusion	Oral
AHU75	*Euphorbia abyssinica *Gmel.	Euphorbiaceae	Dharkena (Or)	TT	Stomachache, malaria Poor sight, paralysis	NN	SyrupSyrup	OralOral
AHU29	*Euphorbia burgeri *M. Gilbert^*♣*^	Euphorbiaceae	Hadami (Or)	Sh	Swollen body (with oozing pus)	L	Crushed & tied	Dermal
AHU35	*Grewia bicolor *Juss.	Tiliaceae	Haroresa, Suta neqebu (Or), Aroresa (S)*	Sh	Stomach disease/worms Intestinal infection Laxative	LBB	Concoction Concoction Concoction	Oral Oral Oral
AHU37	*Grewia ferruginea *Hochst. ex A. Rich.	Tiliaceae	Bururi, Tatesa, Ogemdi gurati (Or), Lato (S)*	Sh	Kidney infection Intestinal parasite	F R	Concoction Concoction	Oral Oral
AHU36	*Heliotropium aegyptiacum *Lehm.	Boraginaceae	Maadaaris (S)	H	Constipation	R	Concoction	Oral
AHU39	*Hibiscus dongolensis *Del.	Malvaceae		H	Dermal infections	L	Crushed & tied	Dermal
AHU28	*Hydnora johannis *Becc.	Hydnoraceae	Tuka(Or), Likki, Likeh, Dise (S)*	RP	Haemorrhage Diarrhea Swollen body part- GOFLA Wound Mouth infections	RFRRR	Crushed & tied Concoction Crushed & tied Crushed & tied Cooked	External Oral Dermal External Eaten
AHU61	*Indigofera amorphoides *Jaub. & Spach	Fabaceae	Jeere (S)	H	Heart disease	L	Hot infusion	Oral
AHU73	*Jatropha curcas *L.	Euphorbiaceae	Andelmeluc (S)	Sh	Serve as purgative	S	Oil squeezed	Oral
AHU34	*Justcia schimperiana *(Hochst. ex Nees) T. Anders.	Acanthaceae	Dhumuga (Or)	Sh	Swelling at ear (Otitis)	St	Small beads of stem prepared and tie on the neck	External
AHU30	*Mentha spicata *L.	Lamiaceae	Nana-kuti (Or)*	H	Stop extended flow of menstruation Blood pressure	LL	Hot infusion Hot infusion	OralOral
AHU13	*Oncoba spinosa *Forssk.	Flacourtiaceae	Jilbo (Or), Bulisagna (S)**	T	Eye disease Dysentry	FR	Ointment Hot infusion	Eye Oral
AHU42	*Opuntia ficus-indica *(L.) Miller	Cactaceae	Tini (Or)**	Sh	Hair fungus	L	Crushed	Dermal
AHU49	*Ozoroa insignis *Del.	Anacardiaceae	Rukeylu (Or), (Ogol (S), Wugr-adad (S); Garri (Or)	T	Tropical ulcer	R	Crushed & tied	Dermal
AHU45	*Plectranthus cylinderaceus *Hochst. ex Benth.	Lamiaceae	Barbarisha gara (Or)	Sh	Spots on skin-infants Spots on skin on baby	LL	Crushed leaf Dried & crushed	Herbal bath Smoke bath
AHU122	*Portulaca oleracea L. *subsp. *oleracea*	Portulacaceae	Merere hare (Or), Siyo (S)*	H	Gastritis, peptic ulcers Constipation Fungal infection	LLL	Cooked vegetable Cooked vegetable Crushed	EatenEaten Dermal
AHU23	*Sarcostemma viminale *(L.) R. Br.	Asclepiadaceae		Cl	Haemorrhage	L & R	Crushed	Anal
AHU38	*Senna italic *Mill.	Fabaceae		H	Stomachache/worm expel	L	Boiled & drunk	Oral
AHU40	*Senna obtusifolia *(L.) Irwin & Barneby	Fabaceae	Jacjacle (S)	Sh	Snake bite	R	Crushed & tied	Dermal
AHU46	*Tamarindus indica *L.	Fabaceae	Roka (Or, S), Hamer (S)**	T	Stomachache/parasite Malaria Dysentery Wound Hemorrhoids Fever	F, SP S, P P, Fl PL, F	Concoction Concoction Powder, concoction Crushed and tied Crushed Concoction	Oral Oral Oral External External, anal Oral
AHU2	*Trichilia emetica *Vahl	Meliaceae	Ununu (Or)	T	Vomiting (Emesis)	B	Concoction	Oral
AHU50	*Withania somnifera *(L.) Dunal	Solanaceae	Hidi gudeye (Or), Guryo-fan (S)	H	Evil eye	L, St	Dried branches	Smoke bath
AHU82	*Ziziphus spina-christi *(L.) Desf.	Rhaminaceae	Kurkura (Or); Gob, Geb (S)**	T	Swollen body part- GOFLA Diarrhea	LL	Concoction Concoction	Herbal bath Oral

**Habit**: Sh-shrub, T-tree, Cl-climber, H-herb; RP-root parasite of trees; Part Used (**PU**), L-leaf, B-bark, St-stem, G-gum, Sa-sap, J-jel, R-root, Fl-flower, F-fruit, S-Seed, P-Pulp, OS-Oil from seed and N-nectar; Method of Preparation (**MP**); Part Administered (**PA**)**/**Method of Application (**MA**).

The distribution of medicinal plant species per growth habit showed that shrubs were 19 (37%) species, trees 15 (29%) species, herbs 13 (26%) species, climbers 3 (6%) species, and root parasite of trees 1 (2%) species. The trees and shrubs constitute more than 60% of the traditional medicinal plants. This can be related with the floristic composition of the vegetation of the area, which is dominated by the *Acacia-Commiphora* woodland, semi-desert scrubland and evergreen scrub vegetation types. The woodlands and the montane vegetation including grasslands and forests and evergreen scrubs and rocky areas contain more medicinal plants with higher concentrations in the woodlands [8].

Among the medicinal plants identified in this study, various parts of 17 species were reported to be edible and hence considered nutraceutical plant species. Thirteen of these nutraceuticals were among the wild edible plants of Fentale area [29]. Among the nutraceutical plants of the present study area, the fruits of *Balanites aegyptiaca, B. glabra, Oncoba spinosa, Opuntia ficus-indica, Tamarindus indica,* and *Ziziphus spina-christi* are marketed in the open local market places.

**Figure 2:**
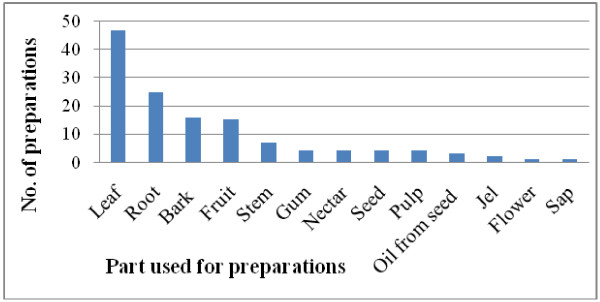
Number of preparations per plant part used.

The informants reported 133 different preparations made from the medicinal plant species. These were cited in the traditional healing system for use in 54 different human ailments. Medicines made from leaves accounted for 47 (35.3%) and roots for 25 (18.8%) of the total preparations (Figure 2).

Inspection of the results on number of preparations and plant parts used may lead to the conclusion that harvesting medicinal plants for use in traditional medicine is not destructive to the natural vegetation of the study area since leaves are the most frequently sought parts of the plant. On the other hand, it may also lead to the conclusion that harvesting of medicinal plants is likely to be destructive because the second most frequently used part is the root. While the traditional healers are collecting the root of the medicinal plants they may affect the whole part. Medicinal plants that are harvested for their roots, rhizomes, bulbs, bark, stem and whole part have severe effects on their survival [6]. Using the roots might be destructive for shrubs and trees under the objective conditions of the study area because 25.3% of the preparations were made from roots of the plants.

### Informants’ consensus factor, frequency of citation and preference ranking

Informants’ consensus factor (F_ic_) was done for the top five disease categories of the study area. The F_ic_ is higher for gastrointestinal disorders and parasites (0.70) and lowest for haemorrhage (0.44) (Table 2).

**Table 2:** Informants’ consensus factor for some common disease categories

**No.**	**Type of disease**	**F**_**ic**_
1	Gastrointestinal disorders and parasites	0.70
2	Wound, Tropical ulcer & swollen body part- GOFLA	0.67
3	Malaria and fever	0.61
4	Mouth, teeth and throat infections	0.52
5	Haemorrhage	0.44

The highest value of F_ic_ was for gastrointestinal problems indicating that there is relatively high consensus on the treatment of gastrointestinal problems with the medicinal plants of the area. Values lower than 0.60 as in haemorrhage and mouth, teeth and throat infections indicate relatively low consensus of informants on those health problems. A similar analysis found high value of F_ic_ for gastrointestinal illness by *Maya, Nahua* and *Zapotec* cultural communities in Mexico [26], indicating that there is relatively high consensus regarding the treatment of gastrointestinal illness.

Medicinal plants that are more popular and widely used by the local community have been prioritized based on frequency of citations. Species that received more than 75% informant report are given in Table 3. For these species, the results show high frequency of citations ranging from 76–96. This shows that there is a considerable level of agreement on the therapeutic worth of these species in the study area. The greater independent citations a particular species receives for the treatment of a certain illness category, the greater is its cultural importance [30].

**Table 3:** List of ten medicinal plants cited by more than 75% of the informants

**Scientific name**	**Ailment**	**No. of informants**	**% of total**
*Aloe pirottae*	Tropical ulcer, Eye disease, Colon problem (SIBRI), Malaria, Snake bite, Gallstone and Insect repellent	48	96
*Azadirachta indica*	Malaria, Fungal infection, Intestinal worms	47	94
*Hydnora johannis*	Haemorrhage, Diarrhoea, Swollen body part (GOFLA*),* Wound, and Mouth infections	45	90
*Tamarindus indica*	Stomachache/parasite, Malaria, Dysentery, Wound, Fever and Haemorrhoids	44	88
*Balanites aegyptiaca*	Snake bite, Premature ejaculation, Influenza, Malaria and Wound	42	84
*Acacia albida*.	Stomachache, diarrhoea, Haemorrhage, Cough, pneumonia, Postpartum complications and Kidney disease	41	82
*Portulaca oleracea L. *subsp. *oleracea*	Gastritis, peptic ulcers, Constipation, and fungal infections	40	80
*Acacia nilotica*	Mouth infection, Toothache, Bad breath (Halitosis), Dysentery and Haemorrhoids	39	78
*Acacia senegal*	Eye disease, Backbone pain, Constipation and Stomachache	38	76
*Asparagus leptocladodius*	Kidney & liver disease, Vomiting of children (Emesis)	38	76

There was repeated mention of the extract from *Aloe pirottae* for use to treat tropical ulcer, eye diseases, malaria, snake bite, gastro-intestinal parasites, gallstone and the jel is dried and ground to produce a product locally known as SIBRI in the Oromo language, which is used for cleaning the human colon. The reports also showed that the leaf, seed and flower of *Azadirachta indica* were concocted and used against malaria, fungal infections and intestinal worms while the root of *Hydnora johannis* was used to treat wound, haemorrhage, diarrhoea and painful body swelling, locally known as GOFLA in the Oromo language.

The higher frequency of citation of these species shows the importance of the species for the local communities and attracts more attention for their conservation in the study area. The depth of medicinal plant knowledge of the age category ranging from 20 to 35 was low (binomial test, p = 0.006) whereas for the age category that ranged above 50 it was rich (binomial test, p = 0.001). The elderly people in the age category above 50 had a much more profound knowledge on the type and use of medicinal plants than the young age category (20–35). There is a significant difference in depth of knowledge between age category ranging from 20 to 35 and age category above 50 (p > 0.05). Here we can see the level of deterioration of indigenous knowledge on medicinal plants in the study area. This might initiate wide scale ethnobotanical study in the area for in-depth investigation of indigenous knowledge that could be followed up with phytochemical and pharmacological analyses in order to give scientific ground to the ethnomedicinal knowledge.

The gender distribution of medicinal plant knowledge showed that men have much more profound knowledge than women (binomial test, p = 0.001). This might be related with the local tradition of restricting traditional medical practices mostly to men as it is also largely true for many other parts of Ethiopia.

Preference ranking value obtained based on the degree of efficacy didn’t show a significant correlation (Spearman correlation test, r = 0.188, α = 0.05, p = 0.275) with that of the informant consensus value. Pharmacologically effective remedies are expected to have greater informant consensus [25]. *Aloe pirottae*, *Balanites aegyptiaca* and *Tamarindus indica* are the three leading species for being used as effective remedies against the corresponding ailments (Table 4).

**Table 4:** Preference ranking of most preferred medicinal plant species based on overall effectiveness to treat human ailments

**Species name**	**Respondents (Traditional healers)**															**Score**	**Rank**
	1	2	3	4	5	6	7	8	9	10	11	12	13	14	15		
*Aloe pirottae*	5	5	5	5	5	5	5	5	4	5	5	5	5	5	5	74	1
*Balanites aegyptiaca*	5	5	5	5	5	5	5	5	4	4	5	4	5	5	5	72	2
*Tamarindus indica*	5	5	5	5	5	5	5	5	4	5	5	4	5	3	5	71	3
*Azadirachta indica*	4	3	5	5	5	5	4	4	5	5	5	5	5	5	3	68	4
*Acacia albida*	5	4	5	5	3	5	5	3	3	5	4	3	5	3	4	62	5
*Hydnora johannis*	5	5	5	3	3	3	5	5	3	5	4	3	4	3	5	61	6
*Portulaca oleracea*	4	3	3	2	3	2	4	3	3	5	4	3	3	3	4	49	7
*Acacia robusta*	5	4	3	3	1	2	3	2	4	5	4	3	2	3	4	48	8
*Withania somnifera*	5	4	2	2	3	2	5	2	3	4	4	3	2	3	4	48	9
*Cucumis dipsaceus*	5	4	3	3	4	2	2	2	3	2	4	3	2	3	4	46	10

Out of these top ten preferred and efficient medicinal plants, some including *Azadirachta indica, Portulaca oleracea* and various species of the genus *Aloes* are also included in the WHO list of most used medicinal plants [31].

A total of 54 different health problems confronting humans were documented in the study area. Accordingly, 15 plant species were used for treatment of gastrointestinal disorders and parasites, 14 species for wound, tropical ulcer and swollen body part including the locally common body swelling known as GOFLA and 8 species for treatment of Malaria and fever (Table 5). The presence of more species for such ailments corresponds to our observation that the problems treated with these plants were the most common health problems of the local communities. According to the informants and data from Babile Wereda (district) health centre, the top common health problems of the locality are internal parasites, diarrhoea, malaria, pneumonia, abdominal pain, dermal infections, eye diseases, infection of skin & subcutaneous tissues. About 81 (71%) of the preparations are made for the most common human health problems of the area.

**Table 5:** Major types of human health problems of the study area and number of plant species used

**No.**	**Type of disease**	**No. of medicinal plants used**	**No. of preparations**
1	Gastrointestinal disorders and parasites	15	24
2	Wound, Tropical ulcer & body swolling - GOFLA	14	15
3	Malaria and fever	8	9
4	Mouth, teeth and throat infections	6	8
5	Haemorrhage	5	5
6	Fungal infection	5	5
7	Snake and carnivore bites	4	5
8	Eye diseases	4	4
9	Kidney infection	3	3
10	Constipation	3	3

In case where different species were prescribed for the same health problem, people showed preference of one to the others. For example, *Acacia robusta, Acokanthera schimperi, Aloe pirottae, Tamarindus indica, Azadirachta indica, Balanites aegyptiaca* and *Euphorbia abyssinica* were all mentioned for use against malaria. However, informants further expressed more preference for the first four species in that order than the others claiming that they are more efficient as medicine for malaria.

### Preparation of medicines and the routes of administration

Local communities employ several methods of preparation of plant material for medicinal use including by crushing, pounding and concocting, which they applied in the form of smoke bath, material for eating raw or just chewing, hot infusion for drinking, fluid extract, decoction, ointment, rubbing, syrup, cream, and others (Table 6). Most of the remedies are prepared from a single species, mixtures are used infrequently. In addition, different plant parts from a single species are prepared in different ways and used to treat the same type of aliment. For example, the fresh leaf and jel extract of *Aloe pirottae* are concocted together and taken orally to treat malaria; the leaf and root of *Sarcostemma viminale* are crushed together and put at the tip of the anal opening to treat haemorrhoid. A number of sources [3,28] reported similar results stating that a single medicinal plant species was used more frequently for remedy preparations, and that mixtures were used rarely in their respective study sites.

**Table 6:** Preparation methods of medicinal plants for use by people

**Method of preparation**	**Total species**	**Percentage**
Crushed & pounded	21	31.8
Concoction	15	22.7
Dried for smoke bath	6	9.1
Fresh parts collected to be eaten raw	5	7.6
Hot infusion	5	7.6
Fluid extract	3	4.5
Decoction	2	3.0
Ointment	2	3.0
Small cuts of fresh root to be rubbed	2	3.0
Syrup	2	3.0
Small cuts of fresh parts to be chewed	1	1.5
Cream	1	1.5
Others	1	1.5

For application of the medicines both internal (52.9%) and external (47.1%) routes were used. The most common route for internal application is oral that accounted for 42.5% and that of external was dermal which accounted for 26.4% (Table 7).

**Table 7:** Route of administration and percentage

**Internal**	**No. of preparation**	**%**	**External**	**No. of preparation**	**%**
Oral	37	42.5	Dermal	23	26.4
Anal	4	4.6	Smoke bath	8	9.2
Eye	3	3.4	Herbal bath	5	5.7
-	-	-	Chew & spit	3	3.4
Others	2	2.3	Others	2	2.3
**Total**	46	52.9	**Total**	41	47.1

The dose given to the patient depends on age, physical and health conditions. Lack of uniformity among informants regarding the doses recommended for certain remedies during prescription and treatment was infrequently observed. For example, one cup of crushed roots of *Acacia robusta* for the treatment of malaria was considered a full dose for many informants, whereas few informants reported that the same amount was administered for three consecutive days as full dose. The results showed that there is lack of precision of the dose in the study area. Especially, in relation to the route of administration where oral application took the lead, lack of precision can be a major drawback. Lack of precision or specifying the dose in rough measurements is a major drawback of application of traditional medicinal plants [28].

## Conclusion

Obviously, herbal medicines are very important in the study area which is an integral part of their culture; and also modern medicine is unavailable and unaffordable for most people. In the study area, the pastoral and agro-pastoral communities maintain and widely use indigenous knowledge on traditional medicinal plants. However, the traditional system and religious beliefs that restrict the manner in which indigenous knowledge should be transferred to others may, with time, lead to the declining of information on medicinal plants as revealed by the trend in this study. It was observed that many young people in the study area are less knowledgeable about the variety and value of indigenous medicinal plants. Therefore, documentation of the knowledge and use of the traditional medicinal plants in the present study area and beyond is very valuable and needs to be scaled-up.

Some of the medicinal plants are also used as wild edible plants. Such benefits of plants as medicine and food (nutraceutical) can be considered as good opportunity for their community based conservation and management. There is a further need for planning of their domestication for better care and enhanced use of the selected species. Most authors who worked on traditional medicinal plants in Ethiopia reported that many plants used in traditional medicine were harvested from the natural stands. This study also showed that the natural stand of the Erer Valley has a huge potential for developing the overall knowledge including the uses of the traditional medicinal plants. It is also the shelter for many plant species, which the local communities make use for fuel, construction, household utilities, market value, flavouring, cleansing, farm tools, honey collection, and so on. In its totality, the Erer Valley is a reserve of botanical wealth. The indigenous knowledge comes from two major ethnic communities (Oromo and Somali) that have a long history of plant use and manipulation and exchange of knowledge as they lived intermixed sharing valuable cultural assets.

However, like else where in the country, nowadays the natural stands of the Erer Valley are becoming negatively impacted as a result of agricultural expansion, livestock population pressure and human settlement. Medicinal plant species of the woodland (for example *Hydnora johannis)* are highly threatened and that might be related to the decline in the population of host trees such as *Acacia nilotica* and *Acacia tortilis.* Visual inspection shows that woodland trees including *Balanites eagyptiaca* and *B. glabra* are locally threatened due to selective cutting of the mother trees for charcoal making. The riverine tree species, *Acacia robusta* and *Tamarindus indica,* are locally threatened due to selective cutting for charcoal making and house construction given the increasing population in the Erer Valley. Along with the loss of the natural vegetation of the valley we are bound to loose the huge potential of the medicinal plants and the associated knowledge. Therefore, the study area calls for urgent measures to be taken to rehabilitate and conserve the remaining vegetation with special regard to the key medicinal plants and preserve the indigenous knowledge.

## Abbreviation

ETH: The National Herbarium of Ethiopia (standard acronym as given in Index Herbariorum, 1990).

## Competing interests

We declare that we don’t have competing interests.

## Authors’ contributions

AB carried out the field study, analyzed the data and wrote the manuscript. ZA revised the manuscript critically and made considerable input for it to have the present form. SD identified the medicinal plant species and did the botanical analysis. NB carried out the field study with AB and organized the data. We all read the final manuscript and agreed on its submission.
